# Supplementary material for: Conformation of the Intermediates in the Reaction Catalyzed by Protoporphyrinogen Oxidase: An In Silico Analysis
Source: Int J Mol Sci. 2020 Dec 14;21(24):9495. doi: 10.3390/ijms21249495 (PMC7764921; doi:10.3390/ijms21249495)
Supplement: Supplementary file 1 [file ijms-21-09495-s001.pdf]

Supplemental Figure S1. Gridbox defining the binding domain of the tetrapyrroles in PPO, including FAD forming the 'roof' of the cavity, the top of α-8 helix of PPO upon which the tetrapyrrole rings are centered at the 'bottom' of the cavity, and arginine 97 involved in stabilizing the rings on the right side of the cavity.

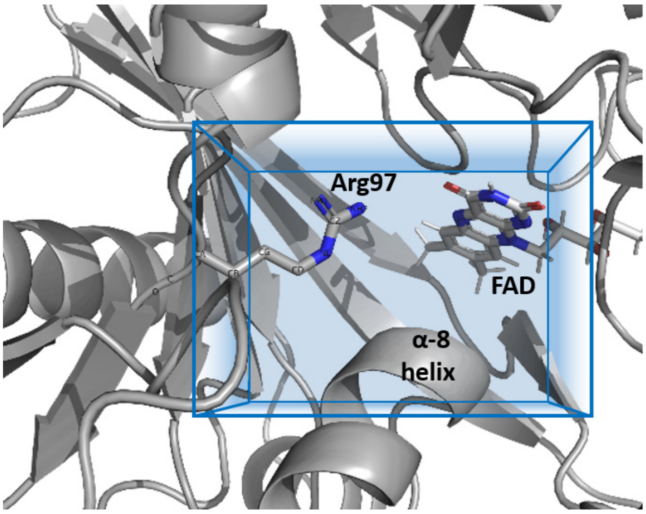

Supplemental Figure S2. Docking poses of the top cluster of intermediate 1a. Top = from above the catalytic domain of PPO and bottom = from inside the catalytic domain of PPO.

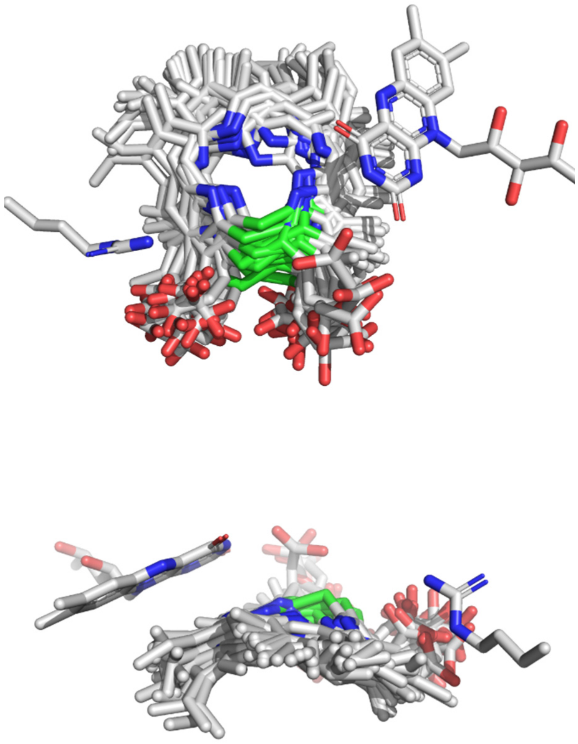

Supplemental Figure S3. Docking poses of the top cluster of intermediate 1c. Top = from above the catalytic domain of PPO and bottom = from inside the catalytic domain of PPO.

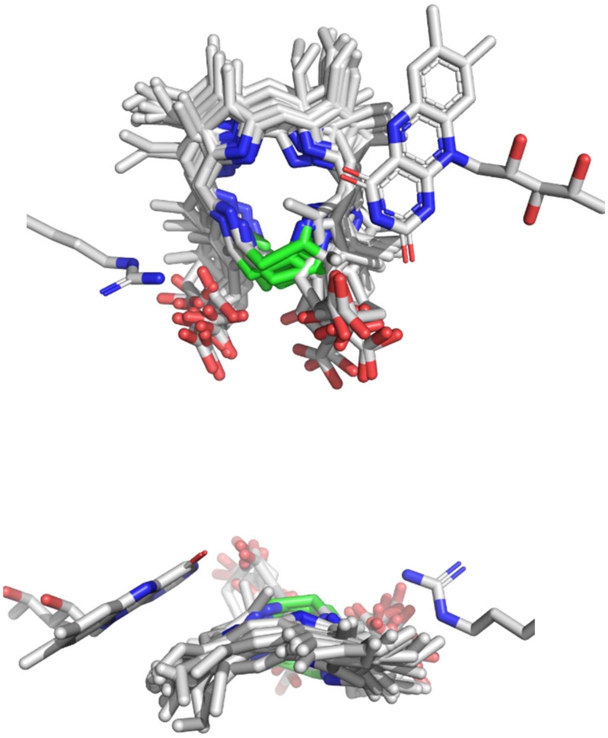

Supplemental Figure S4. Docking poses of the top cluster of intermediate 2a. Top = from above the catalytic domain of PPO and bottom = from inside the catalytic domain of PPO.

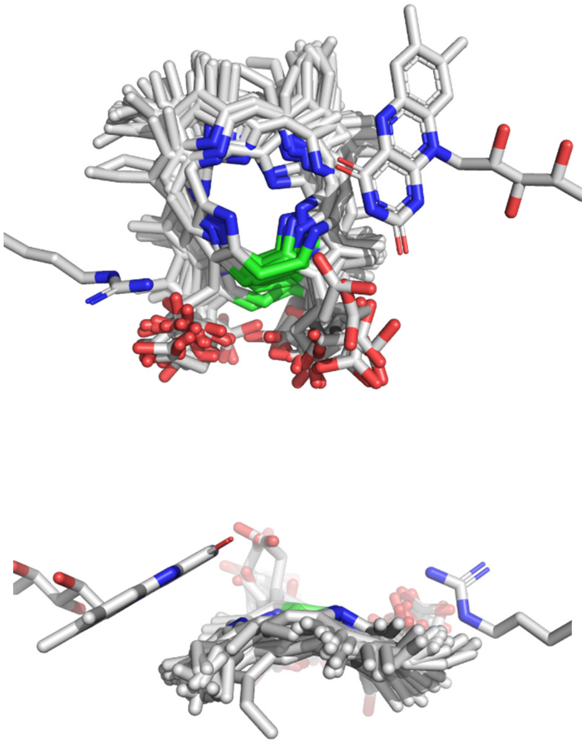

Supplemental Figure S5. Docking poses of the top cluster of intermediate 2d. Top = from above the catalytic domain of PPO and bottom = from inside the catalytic domain of PPO.

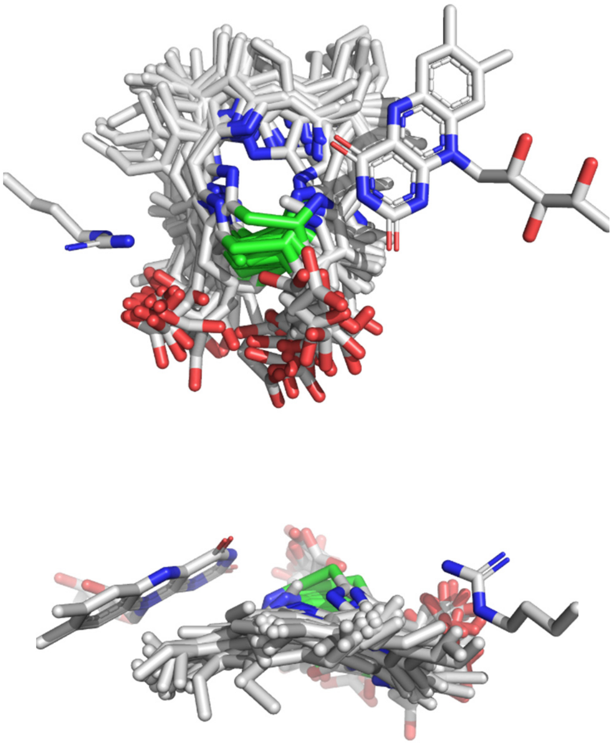

Supplemental Figure S6. Docking poses of the top cluster of intermediate proto. Top = from above the catalytic domain of PPO and bottom = from inside the catalytic domain of PPO.

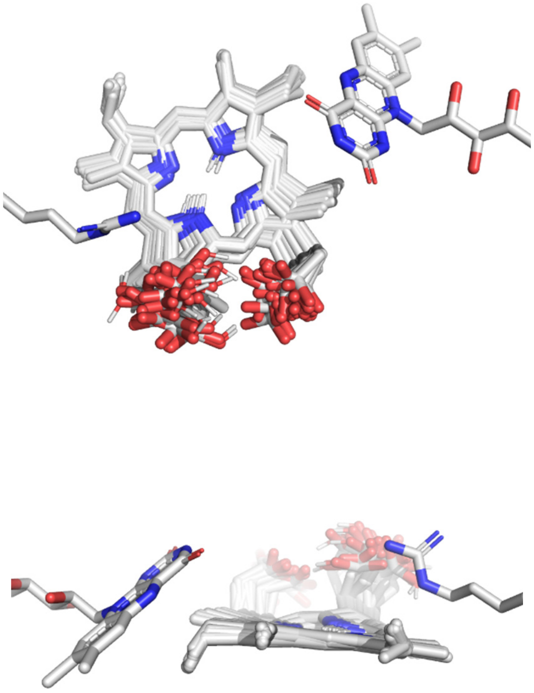

Supplemental Figure S7. DFT calculations on protoporphyrinogen IX pyrroles 1 and 2 in equilibrium geometry with DFT wB97XD 6-31G*.

DFT calculation on protoporphyrinogen pyrrole 1
Equilibrium geometry with DFT wB97XD 6-31G*
Neutral molecule

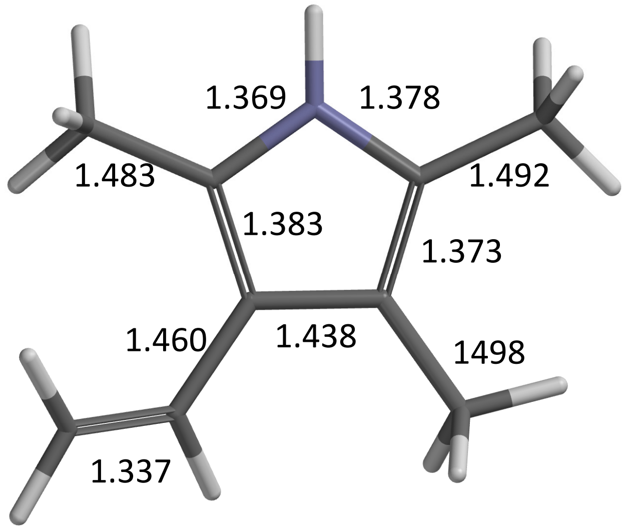

DFT calculation on protoporphyrinogen pyrrole 2
Equilibrium geometry with DFT wB97XD 6-31G*
Carrying negative -1 charge

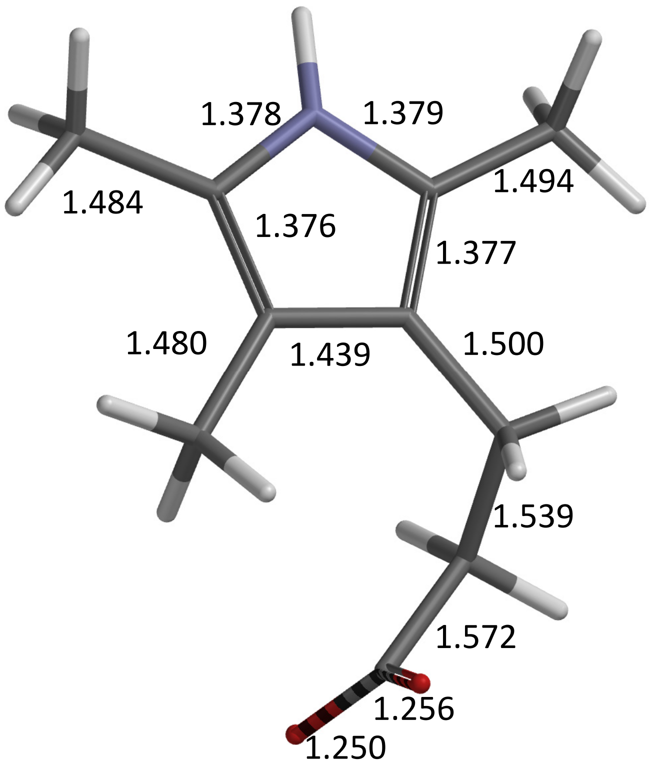

Supplemental table S1. Percent identity matrix of the Clustal Omega alignment made with the file of 16 model organisms protoporphyrinogen oxidase proteins.

		1	2	3	4	5	6	7	8	9	10	11	12	13	14	15	16
1	PPO C. elegans	100	12.96	12.7	13.19	13.35	14.91	14.95	13.71	13.82	15.25	17.7	10.66	11.85	12.89	13.84	14.29
2	PPO Saccharomyces cerevisiae	12.96	100	27.33	22.88	24.58	26.11	24.09	24.09	19.87	20.73	20.26	21.88	19.26	21.7	21.67	20.35
3	PPO2 Drosophila melanogaster	12.7	27.33	100	36.01	40.43	37.96	38.48	38.04	25.56	25.11	24.72	22.81	29.48	22.37	23.94	22.65
4	PPO Trichoplax sp. H2	13.19	22.88	36.01	100	39.66	40.64	40.17	39.74	27.88	28.32	26.83	27.42	28.76	24.89	25.22	26.55
5	PPO2 Danio Rerio	13.35	24.58	40.43	39.66	100	57.89	52.85	50.74	24.16	25.28	25.11	27.15	31	25.84	23.73	24.72
6	PPO2 Anolis carolinensis	14.91	26.11	37.96	40.64	57.89	100	58.02	57.17	25.28	24.83	26.01	28.73	32.35	24.05	25.5	24.28
7	PPO2 Homo sapiens	14.95	24.09	38.48	40.17	52.85	58.02	100	88.68	24.94	26.06	24.55	25.96	30.86	25.94	27.15	25.5
8	PPO2 Mus musculus	13.71	24.09	38.04	39.74	50.74	57.17	88.68	100	24.94	24.72	25	25.96	31.08	26.39	26.93	25.72
9	PPO2 Oryza sativa japonica	13.82	19.87	25.56	27.88	24.16	25.28	24.94	24.94	100	57.75	57.26	22.3	27.45	26.57	26.85	27.29
10	PPO2 Arabidopsis thaliana	15.25	20.73	25.11	28.32	25.28	24.83	26.06	24.72	57.75	100	66.53	24.12	26.95	27.29	26.57	27
11	PPO2 Nicotiana tabacum	17.7	20.26	24.72	26.83	25.11	26.01	24.55	25	57.26	66.53	100	23.5	26.33	27.94	26.79	27.97
12	PPO Bacillus subtilis	10.66	21.88	22.81	27.42	27.15	28.73	25.96	25.96	22.3	24.12	23.5	100	27.68	27.25	30.33	29.71
13	PPO Myxococcus Xanthus	11.85	19.26	29.48	28.76	31	32.35	30.86	31.08	27.45	26.95	26.33	27.68	100	29.51	29.36	30.04
14	PPO Chlamydamonas reinhardtii	12.89	21.7	22.37	24.89	25.84	24.05	25.94	26.39	26.57	27.29	27.94	27.25	29.51	100	56.32	57.76
15	PPO1 Arabidopsis thaliana	13.84	21.67	23.94	25.22	23.73	25.5	27.15	26.93	26.85	26.57	26.79	30.33	29.36	56.32	100	75.99
16	PPO1 Nicotiana tabacum	14.29	20.35	22.65	26.55	24.72	24.28	25.5	25.72	27.29	27	27.97	29.71	30.04	57.76	75.99	100
